# Five years after introduction of an opt-out consent system for organ donation; lessons learned from England and the Netherlands

**DOI:** 10.3389/ti.2026.16664

**Published:** 2026-09-15

**Authors:** Nichon Esther Jansen, Tineke Jentina Wind, Martin B. A. Heemskerk, Aline C. Hemke, Claire Williment, Sue Madden, Dale Gardiner

**Affiliations:** 1 Research Department, Dutch Transplant Foundation, Leiden, Netherlands; 2 Department of Organ Donation Coordination, Maastricht Universitair Medisch Centrum+, Maastricht, Netherlands; 3 NHS Blood and Transplant Blood Donation, Bristol, United Kingdom

**Keywords:** donor register, England and Netherlands, lessons learned, opt-out consent system, organ donation policy

## Abstract

Five years after changing from opt-in to opt-out consent systems in England and the Netherlands, we examine the impact on donor registrations, consent rates, donor numbers, and reflect on lessons learned. Opt-out legislation was implemented in May 2020 in England and July 2020 in the Netherlands. In England, 44% of the population is now registered; 40% recording an opt-in decision and 4% an opt-out decision. In the Netherlands, mandated donor registration resulted in the entire adult population having their decision recorded: “Yes, I want to be a donor” (34%), “No objection” (24%), or “No, I don’t want to be a donor” (31%), while 11% leave the “Decision to next of kin”. Despite these structural changes, the consent categories created by opt-out legislation (“deemed” consent in England and “No objection” in the Netherlands) remain challenging in practice, with fewer than 50% of families agreeing to donation. In England, the family consent rate declined from 69% to 57% between 2020 and 2025 and the donor numbers remain below pre-pandemic levels. In contrast, the Netherlands experienced a modest increase in family consent rates and donor numbers compared to the opt-in consent system. Opt-out legislation provides an important policy framework, but sustained system-level commitment remains essential.

## Graphical Abstract

**Figure:**
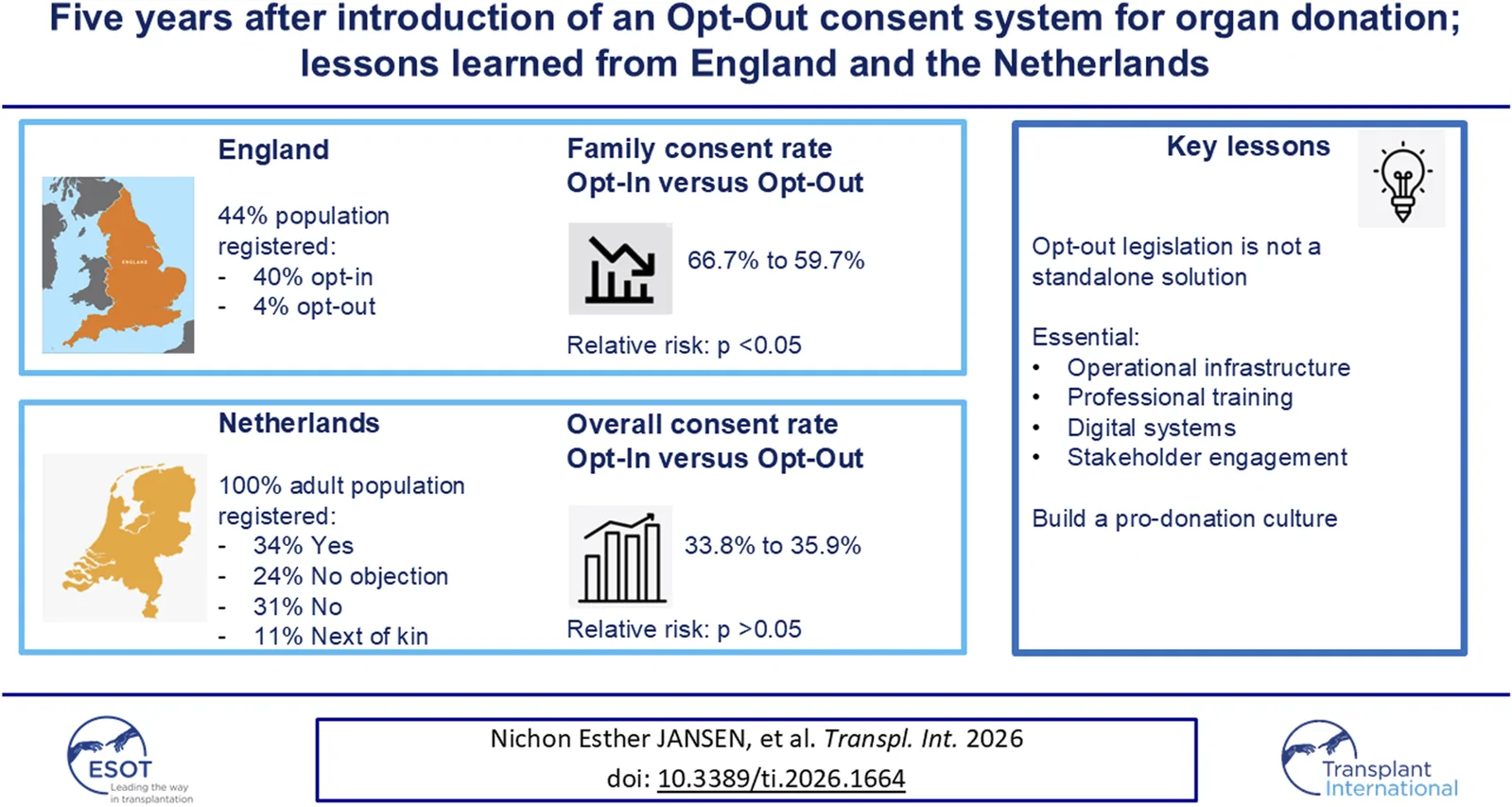

## Introduction

In May and July 2020, England and the Netherlands shifted their consent systems for organ donation from Opt-In to Opt-Out. The primary aim was to increase consent for organ donation and, ultimately, increase the number of organ donors.

In England, all adults aged 18 and over are considered potential organ and tissue donors unless they have made a decision not to donate or are part of a small number of protected groups. Under the legislation this is referred to as deemed consent. Within the United Kingdom, Wales commenced deemed consent in 2015, England 2020, Scotland in 2021 and Northern Ireland in 2023. The legislation across the four UK nations, and the UK’s Crown Dependencies, is broadly similar [1].

While there is no legal requirement to record a donation decision, individuals may choose to register an opt-in or opt-out decision on the National Health Service (NHS) Organ Donor Register (ODR) or express their wishes verbally to family members, who can relay this information during donation discussions.

The primary route for opt-in registrations is through driving licence applications or renewals, accounting for approximately 89% of registrations [2]. In comparison, around 7% occur via the website and 4% via the NHS App. Opt-out decisions can only be recorded through the website or NHS App. In the Netherlands, an “active donor registration” process was implemented, fundamentally changing the structure of the Donor Register into a mandated choice system. By July 2021, the entire population aged 18 and older had a recorded decision. Individuals could register “Yes,” “No,” or delegate the decision to next of kin or a specific person. Those who did not respond were registered as having “No objection,” ensuring a recorded preference for all adults. Individuals can change their decision at any time. In 2022, several of the current authors (NEJ, CW, DG) published an early comparative analysis of the introduction of opt-out legislation in England and the Netherlands [3]. Full details of implementation and the consent systems are outlined in that paper. We concluded that legislation alone is unlikely to increase donation and that successful implementation requires established operational infrastructure, adoption of digital infrastructure, clinical codes of practice, professional training, sustained public awareness campaigns and engagement across society, including with faith and community groups.

We also observed that legislative reform can enable wider system investment, stimulating funding for donation infrastructure and public promotion, and opening dialogue with communities about organ donation. At the time of publication, however, it was too early to determine the impact of opt-out legislation on consent rates or donor numbers, and any early analysis would be confounded by the COVID-19 pandemic.

Now, 5 years after the introduction of opt-out legislation, this study evaluates its impact on donor registrations, consent rates and donor numbers in England and the Netherlands, and provides further insights into the lessons learned.

## Materials and methods

### England

Data on donor registrations, consent rates and numbers of organ donors were obtained from NHS Blood and Transplant (NHSBT), which maintains the ODR, the Potential Donor Audit (PDA) and the UK Transplant Registry.

The PDA captures all deaths under the age of 81 occurring in UK intensive care units or emergency departments and identifies potential organ donors. Data are entered by Specialist Nurses in Organ Donation (SNODs). A key metric derived from the PDA is the organ donation consent rate, defined as the proportion of eligible families approached for a donation decision in whom consent for donation is obtained; in England this includes those donors who have registered an opt-out decision. For comparative purposes in this paper, this is referred to as family consent. Population estimates for England were obtained from the Office for National Statistics [4].

### The Netherlands

In the Netherlands, donor registration data were obtained from the national Donor Register [5]. In addition, medical records of patients who died in intensive care units were reviewed retrospectively. Potential donors were defined as donors who are medically suitable for donation, regardless of their registration in the Donor Register, including those with a “No” registration. In the Netherlands the key measure of consent is the overall consent rate, defined as the proportion of all potential donors in whom consent for donation is obtained; this includes those who have registered a “No” decision.

These data are recorded in NovaNORD, a database maintained by the Dutch Transplant Foundation, and entered by (organ) donor coordinators [6].

### Comparative analysis

For comparative purposes, donor register data for both countries were summarised for the period before and after the introduction of opt-out legislation (2019–2025). Organ donation consent rates were analysed for 2017–2025 and deceased donor numbers for 2010–2025 to provide longer-term contextual trends.

Relative Risk and corresponding 95% confidence intervals (CI’s) were calculated to test the hypothesis that opt-out legislation would increase the overall consent rate for organ donation. The “Risk in exposed group” is analysed for the years *during* the opt-out legislation (2022–2025) and the “Risk in unexposed group” for the years *before* implementation of the legislation (2017–2019). The years 2020 and 2021 were excluded due to potential confounding effects of the COVID-19 pandemic. Statistical significance was assessed using P-values corresponding to values of the standard normal distribution; a P-value <0.05 was considered statistically significant. All Sankey diagrams created using SankeyMATIC.

### Ethics statement

This study, using data from Dutch and English organ donation data registers, was conducted in accordance with the principles of the World Medical Association Declaration of Helsinki. Both the Netherlands and England operate under ethical frameworks consistent with the Declaration of Istanbul on Organ Donation and Transplantation. No interventions or procedures involving human participants were performed, and only anonymized, registry-based data were used.

## Results

### Organ donor registrations

#### England

In January 2026, 44% of the 57.7 million residents in England were registered on the ODR, comprising 40% with an opt-in decision and 4% with an opt-out decision. This compares to 35% opt-in and 2% opt-out of the population in January 2020, immediately prior to the implementation of deemed consent legislation. Only 136 individuals had nominated a representative to make a donation decision on their behalf. Trends in donor registration between 2019 and 2026 are shown in Figure 1.

**FIGURE 1:**
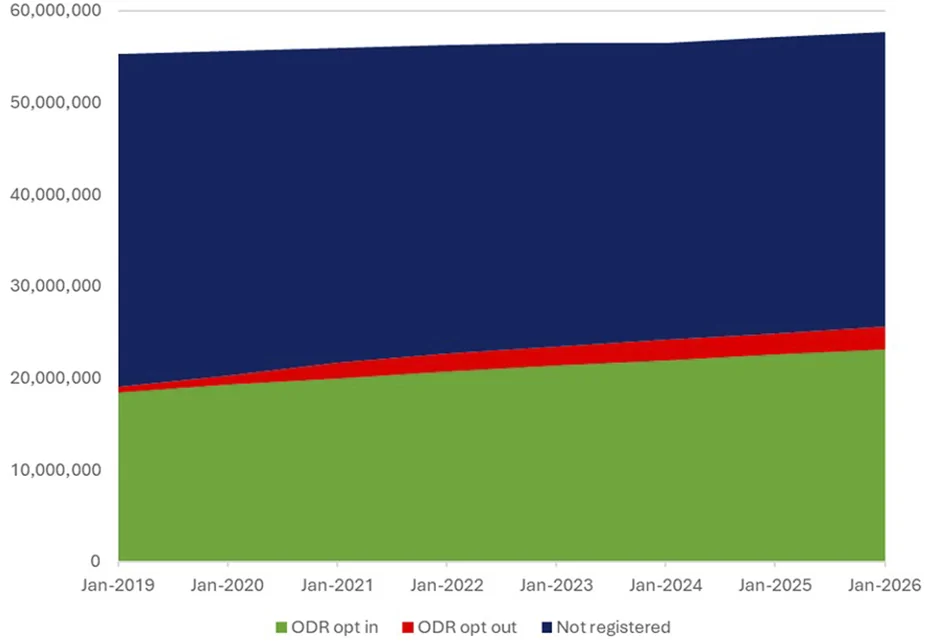
Number of donor registrations in England on the NHS Organ Donor Register 1^st^ of January 2019–2026*. * Only 136 individuals have nominated a representative to make a donation decision on their behalf, so this is not displayed in the figure.

#### The Netherlands

Following the active donor registration process, the entire Dutch population aged 18 years and older was recorded in the Donor Register by July 2021. In January 2026, 34% of residents had registered “Yes, I want to be a donor,” 24% “No objection,” and 31% “No, I do not want to be a donor,” while 11% delegated the “Decision to next of kin” or “Specific person.” Overall, 58% of the population had registrations permitting organ donation. Registration proportions have remained largely unchanged since July 2021 (Figure 2).

**FIGURE 2:**
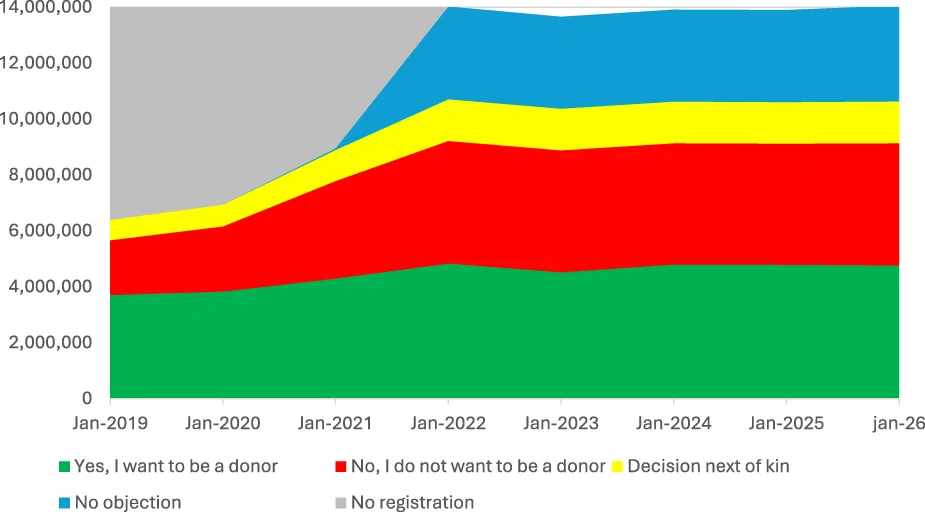
Number of donor registrations in the Netherlands on the 1st of January 2019–2026.

### Family consent rates for organ donation

#### England

The annual family consent rate for organ donation following approaches to donor families in England declined from 69% in 2020 to 57% in 2025, the lowest level recorded in more than a decade (Table 1). See Supplementary Table 2 for the distinction in family consent rates between donation after brain death (DBD) and donation after circulatory death (DCD) donors.

**TABLE 1:** Annual consent rate for organ donation following approaches of donor families in England and the Netherlands January 2017 – December 2025.

Calendar year	England		Netherlands	
​	Opt-out implemented May 20, 2020		Opt-out implemented July 1, 2020	
​	Annual family consent rate (%)	Monthly range (min %, max %)	Annual family consent rate (%)	Monthly range (min %, max %)
2017	65	(62, 69)	45	Not available
2018	67	(61, 71)	44	Not available
2019	68	(64, 74)	42	Not available
2020	69	(65, 74)	48	Not available
2021	66	(58, 72)	60	(52, 71)
2022	62	(56, 70)	60	(51, 65)
2023	61	(57, 70)	61	(49, 72)
2024	59	(52, 62)	59	(48, 73)
2025	57	(54, 61)	56	(48, 61)

In the Netherlands, the family consent rate includes all potential donor families approached for donation. Following the implementation of the legislation, many individuals registered as ‘No' were no longer approached because their donation decision was already known. In England, the family consent rate includes all eligible donor families approached for donation, including those of donors who had opted out.

#### The Netherlands

In the Netherlands, the annual family consent rate for organ donation following approaches to donor families declined from 60% in 2021 to 56% in 2025. However, this is still higher than the family consent rate under the opt-in system, which peaked at 48% in 2020 (Table 1).

### Donor registration, family approach, and overall consent rate

#### England

Changes to the PDA data collection system in September 2020 enabled more accurate recording of donor registration status. Consequently, the proportion of unknown registrations declined from 71% in 2017 to 12% in 2025 (Table 2). Since September 2020, the potential donors with known registration status, the proportion registered as opt-in increased steadily to 37% by 2025, while opt-out registrations remained low at approximately 3%.

**TABLE 2:** Registration on the Organ Donor Register (where known) of identified potential organ donors* in England included in the Potential Donor Audit 2017–2025.

Calendar year	Total audited deaths number	Total potential donors number	Total number of potential donors where ODR registration known	ODR opt-in registration number/Total where ODR registration known number (%)	ODR opt-out registration number/Total where ODR registration known number (%)	Not registered number/Total where ODR registration known number (%)	*Register not consulted or outcome not documented number/Total potential donors number (%)
2017	28,507	6,226	1,786	1,564/1,786 (87.6)	7/1,786 (0.4)	215/1,786 (12.0)	4,440/6,226 (71.3)
2018	27,926	6,196	1,772	1,539/1,772 (86.9)	24/1,772 (1.4)	209/1,772 (11.8)	4,424/6,196 (71.4)
2019	27,631	6,331	1,965	1,720/1,965 (87.5)	42/1,965 (2.1)	203/1,965 (10.3)	4,366/6,331 (69.0)
2020	30,960	6,367	2,743	1,581/2,743 (57.6)	100/2,743 (3.6)	1,062/2,743 (38.7)	3,624/6,367 (56.9)
2021	35,239	6,255	4,707	1,615/4,707 (34.3)	123/4,707 (2.6)	2,969/4,707 (63.1)	1,548/6,255 (24.7)
2022	31,195	5,895	5,035	1,713/5,035 (34.0)	148/5,035 (2.9)	3,174/5,035 (63.0)	860/5,895 (14.6)
2023	29,975	5,937	5,153	1,887/5,153 (36.6)	130/5,153 (2.5)	3,136/5,153 (60.9)	784/5,937 (13.2)
2024	29,888	5,910	5,123	1,871/5,123 (36.5)	139/5,123 (2.7)	3,113/5,123 (60.8)	787/5,910 (13.3)
2025	27,865	5,714	5,024	1,878/5,024 (37.4)	169/5,024 (3.4)	2,977/5,024 (59.3)	690/5,714 (12.1)

* The ODR, is not consulted for all potential donors; where potential donors are assessed to be medically unsuitable for organ donation prior to the family approach, the ODR, register may not be checked.

The consent group of identified eligible organ donors included in the PDA in England 2017 – 2025 is provided in Supplementary Table 1. The number of families approached for organ donation, based on the consent group, and family consent rate in England 2017 – 2025 is given in Supplementary Table 3. The Sankey visuals compare the period before and after implementation of opt-out legislation, see Figure 3.

**FIGURE 3:**
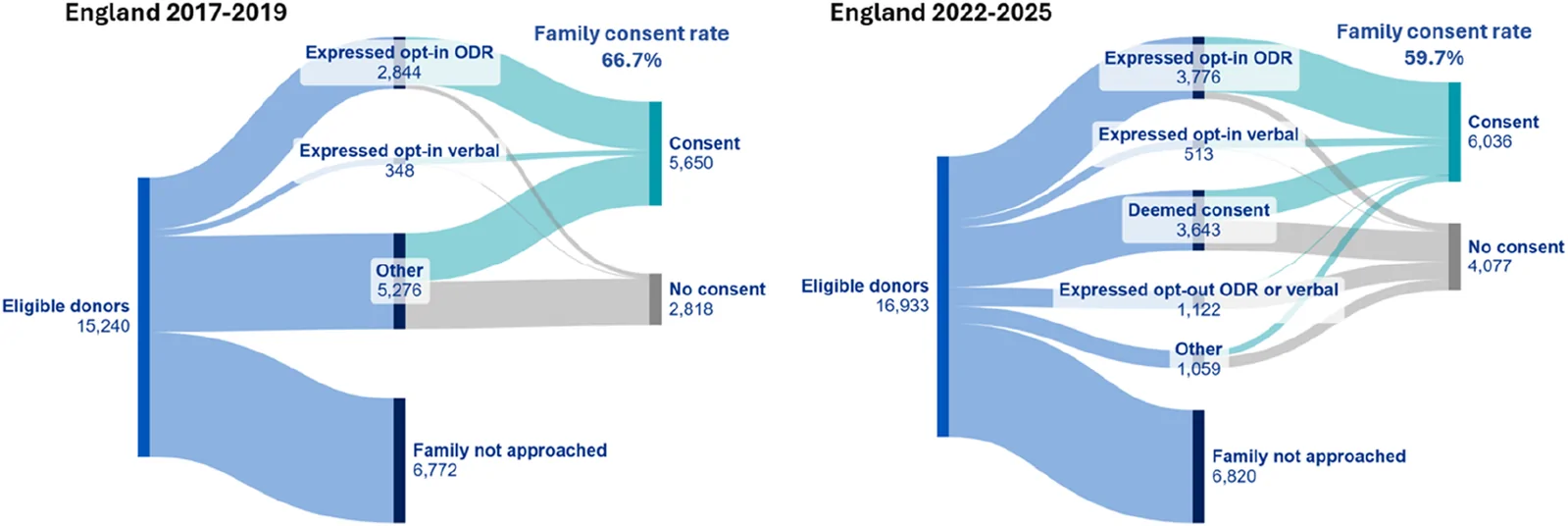
Number of eligible donors, family approaches and family consents by consent group in England, pre and post opt-out legislation. Around a third of eligible donors, in England, are deemed medically unsuitable through donor screening programs so families are not approached. Note: The family consent rate is presented in this diagram, the denominator differs from that used in Figure 4 for the Netherlands.

The inferential statistical analysis, using Relative Risk, showed that the overall consent rate in England is significantly lower following the implementation of opt-out legislation compared to the period before, see Table 3.

**TABLE 3:** Comparison of overall consent rates in England and the Netherlands before and after implementation of the Opt-Out Act.

Hypothesis	Group	Consent (n)	Objection (n)	Total	Risk [95% CI]	Relative risk (RR); [95%CI]	Chi	P-value
England								
Overall consent rate	Exposed (opt-out Act)	6,063	10,897	16,933	0.356 [0.349–0.363]	0.961 [0.934–0.989]	−2.65	<0.05*
	Unexposed (before opt-out Act)	5,650	9,590	15,240	0.370 [0.363–0.378]			
Netherlands								
Overall consent rate	Exposed (opt-out Act)	1,022	2,001	3,023	0.338 [0.321–0.354]	1.060 [0.995–1.128]	1.842	>0.05
	Unexposed (before opt-out Act)	1,679	3,006	4,685	0.358 [0.344–0.372]			

CI , confidence interval; * = p-value <0.05.

#### The Netherlands

Analysis of potential organ donors between 2017 and 2025 shows an increase in “Yes” registrations from 24% to 29% and the emergence of the “No objection” category to approximately 20% of potential donors (Table 4). Over the same period, the proportion of potential donors registered as “No, I do not want to be a donor” increased from 16% to 39%. The proportion without a recorded registration declined from 52% to 6% following implementation of active donor registration.

**TABLE 4:** Registration in the Donor Register of identified potential organ donors* in the Netherlands in the intensive care unit 2017–2025.

Calen-dar year	Total deaths in ICUs number	Total identified potential donors number	Yes, I want to be a donor number/Total registered number (%)	No objection number/ Total registered number (%)	No, I do not want to be a donor number/Total registered number (%)	Decision by next of kin number/Total registered number (%)	Not registered number/Total registered number (%)	*Register not consulted number/Total potential donors number (%)
2017	7,755	951	203/851 (23.6)	NA	137/851 (16.1)	67/851 (7.9)	444/851 (52.2)	100/951 (10.5)
2018	7,990	1,039	229/921 (24.9)	NA	158/921 (17.2)	64/921 (6.9)	470/921 (51)	118/1,039 (11.4)
2019	7,373	1,033	189/904 (20.9)	NA	184/904 (20.4)	62/904 (6.6)	469/904 (51.9)	129/1,033 (12.5)
2020	7,618	987	205/876 (23.4)	NA	204/876 (22.8)	52/876 (5.9)	415/876 (47.4)	111/987 (11.2)
2021	7,750	915	250/874 (28.6)	158/874 (18.1)	302/874 (34.6)	80/874 (9.2)	84/874 (9,6)	41/915 (4,5)
2022	6,981	1,102	309/1,050 (29.4)	214/1,050 (20.4)	403/1,050 (38.4)	79/1,050 (7.5)	45/1,050 (4.3)	52/1,102 (4.7)
2023	6,810	1,147	318/1,101 (28.9)	218/1,101 (19.8)	439/1,101 (39.9)	77/1,101 (7)	49/1,101 (4.5)	46/1,147 (4)
2024	6,793	1,244	344/1,188 (29)	237/1,188 (19.9)	426/1,188 (35.9)	110/1,188 (9.3)	71/1,188 (6)	56/1,244 (4.5)
2025	6,732	1,192	318/1,129 (28.2)	222/1,129 (19.7)	440/1,129 (39)	78/1,129 (7)	71/1,129 (6.3)	63/1,192 (5.3)

NA, not applicable.

* The Donor Register was not consulted for all identified potential organ donors, such as in cases where donation had already been discussed with the family before consulting the register, the donor was from abroad, or the donor was a child.

Family support varied by registration category. Families generally supported donation when the donor had registered “Yes,” whereas in cases of “No objection” families declined donation in more than half of cases (Supplementary Table 4). When the decision was delegated to next of kin, consent rates ranged between 28% and 52%. Considering both donor registration and family decision-making, the overall consent rate across all potential donors remained approximately 35% between 2017 and 2025.

The Sankey visuals compare the period before and after implementation of opt-out legislation, see Figure 4.

**FIGURE 4:**
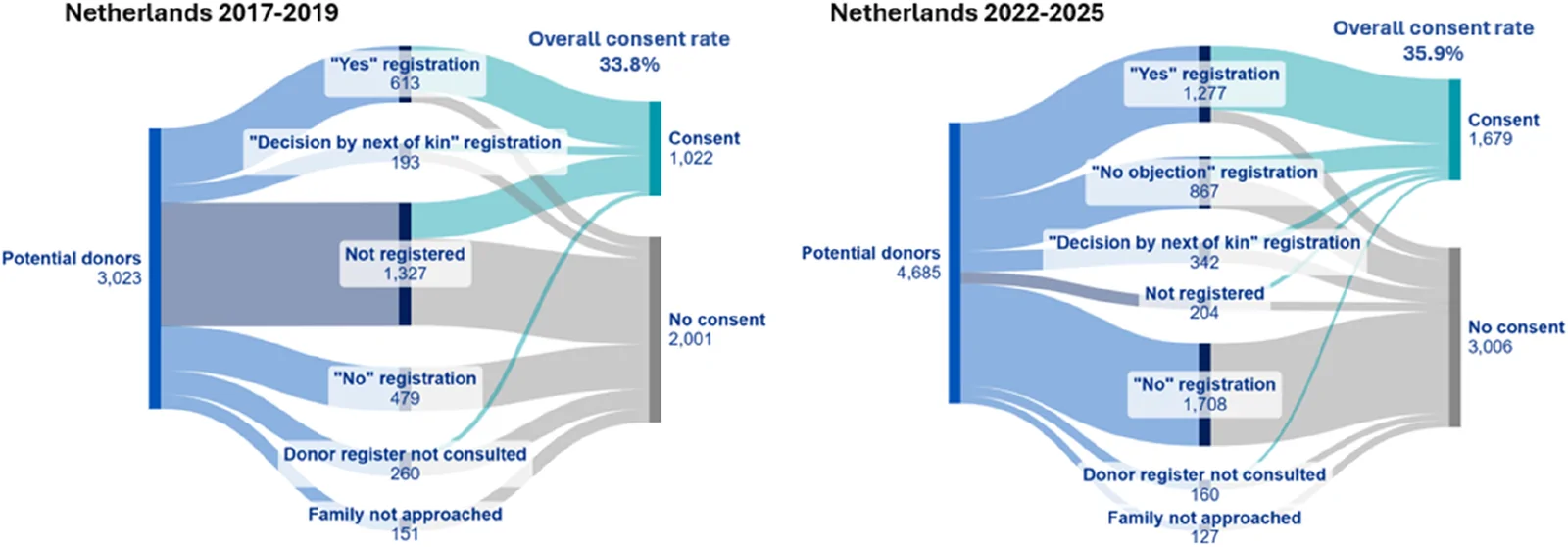
Number of potential donors, family approaches and family consent per outcome in the Donor Register in the Netherlands, pre and post opt-out legislation. Note: This diagram presents the overall consent rate, the denominator differs from that in Figure 3 for England.

The inferential statistical analysis, using Relative Risk, showed that the overall consent rate in the Netherlands is not significantly higher following opt-out legislation compared to the period before, see Table 3.

### Organ donor numbers

#### England

Deceased donor numbers declined sharply in 2020, coinciding with the introduction of opt-out legislation and the COVID-19 pandemic. Donor numbers fell from a peak of 1,432 in 2019 to 1,043 in 2020. Numbers have since increased gradually to 1,207 in 2025, although they remain below pre-pandemic levels (Figure 5). In recent years, DCD has exceeded DBD, with 678 DCD and 529 DBD donors recorded in 2025.

**FIGURE 5:**
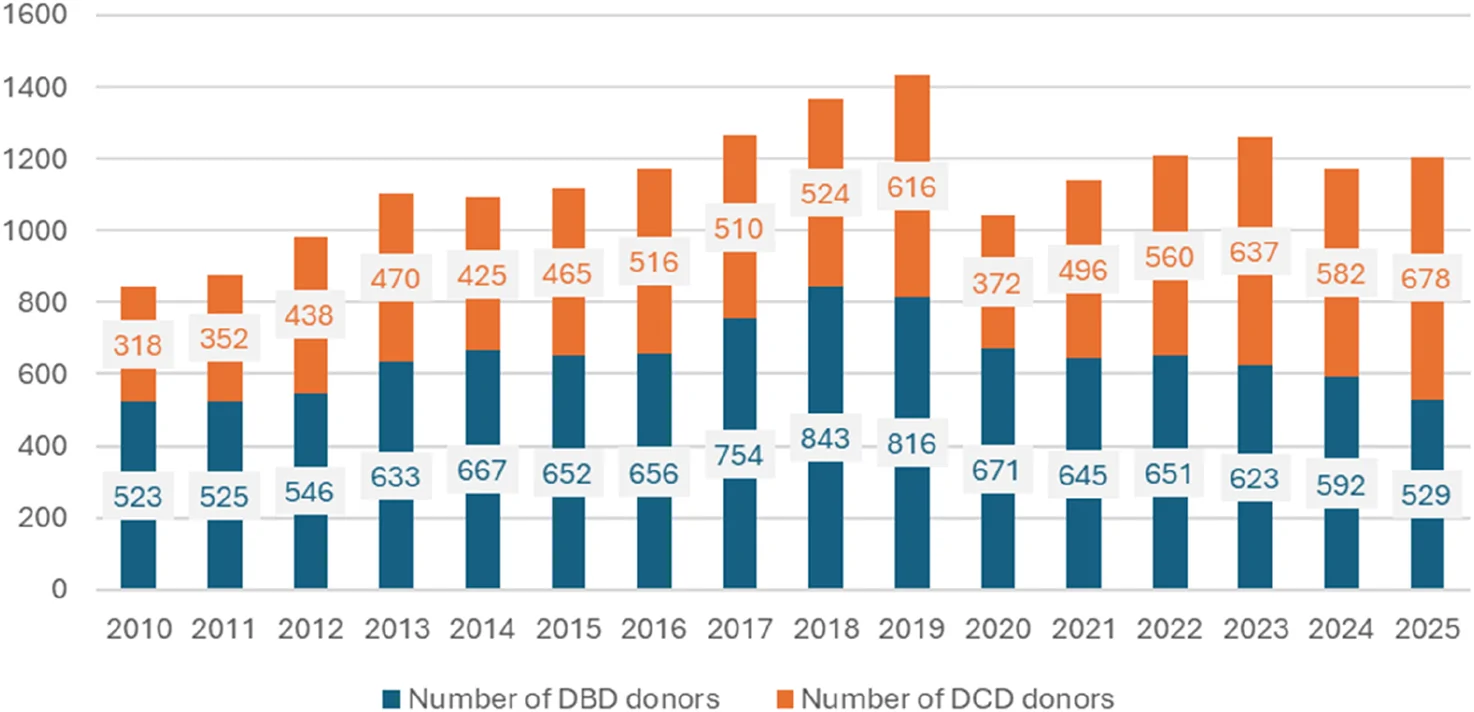
Number of DBD, DCD organ donors in England 2010–2025. Donation after brainstem death (DBD) = deceased organ donation following diagnosis of death using neurological criteria; Donation after circulatory death (DCD) = deceased organ donation following diagnosis of death using cardiorespiratory diagnosis.

#### The Netherlands

Between 2010 and 2025, the number of deceased donors in the Netherlands increased following implementation of the opt-out system, with the highest number recorded in 2024 (n = 360). However, donor numbers in recent years remain broadly comparable with earlier peaks, such as 2014 (n = 269). A notable trend is the increasing number of donors following euthanasia, rising from 13 in 2022 to 34 in 2025. In total, 189 organ donors following euthanasia have been realised since 2012 (Figure 6).

**FIGURE 6:**
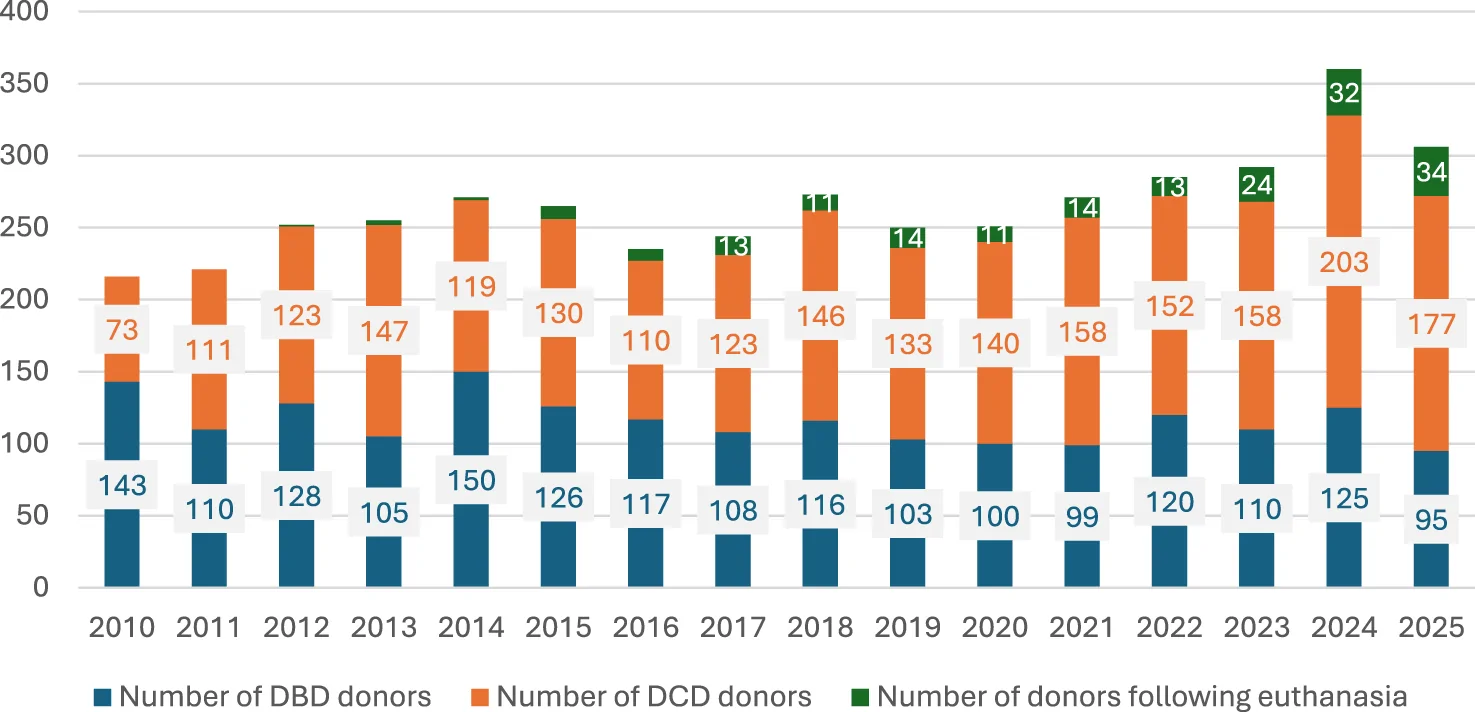
Number of DBD, DCD, and donors following euthanasia in the Netherlands 2010–2025. Donation after brain death (DBD) = deceased organ donation following diagnosis of death using neurological criteria; Donation after circulatory death (DCD) = deceased organ donation following diagnosis of death using cardiorespiratory diagnosis.

## Discussion

### Reflections after 5 years of implementation of the opt-out consent system

#### England

A government-commissioned evaluation of deemed consent legislation in England examined the perspectives of families approached for organ donation and healthcare professionals involved in donation discussions [7, 8]. Many families had limited understanding of deceased organ donation and perceived themselves as the principal decision-makers, rather than deemed consent as an expression of the deceased’s choice. Families relied heavily on specialist nurses in organ donation for guidance and emotional support and reported high satisfaction with their care. Healthcare professionals reported that the legislation had not reduced the complexity of family discussions or end-of-life care, and most remained unconvinced that legislation alone would improve consent rates. The evaluation concluded that deemed consent was unlikely to have caused the observed decline in consent rates, suggesting other factors influenced the reduction in consent and donor numbers.

Public attitudes towards the NHS have shifted markedly since the COVID-19 pandemic. YouGov® polling indicated 70% of respondents rated the NHS as “good” in 2020, whereas since October 2022 more than 60% have consistently rated it as “bad” [9]. A preliminary analysis suggests a moderate positive correlation between positive public opinion of national NHS services and family consent rates (correlation coefficient +0.73; Supplementary Figure 1). Internal NHSBT surveys similarly show declining public awareness of the organ donation system and softening support for donation in principle.

In England, the coexistence of opt-in and opt-out pathways creates a complex dual consent system. Deemed consent may confuse the public, leading to misunderstanding or disengagement, where individuals neither opt-in nor opt-out. Registration patterns have remained relatively stable since implementation, suggesting limited sustained public engagement. On the other hand, the inferential analysis demonstrated significant lower overall consent rates.

England operates a “soft” opt-out model in which families are expected to support the deceased’s decision. In practice, families may override both expressed opt-in decisions and deemed consent. In more than 50% of applicable deemed cases in recent years, families have not supported donation (Table 3). This highlights the continued centrality of the family at the bedside and raises important questions about the practical authority of individual decisions and “soft” opt-out legislation. Similar patterns across other UK nations suggest these challenges are systemic rather than purely legislative.

Together, these findings suggest that legislative reform alone is insufficient to create a supportive culture for deceased organ donation. Societal understanding, trust in healthcare institutions and effective professional practice remain central to donation discussions and family consent.

#### The Netherlands

Since implementation of the opt-out system in July 2020, active donor registration has doubled registrations in the Donor Register from 7 million to 14 million, covering the entire population aged 18 and older. Knowing the potential donor’s wishes provides greater clarity during the family approach. The annual family consent rate rose to approximately 56%, compared with 48% under the opt-in system. However, the large number of “No, I do not want to be a donor” registrations has considerably reduced the donor pool. When the entire consent process, from donor registration to family approach, is considered, the increase in overall consent rate compared with the opt-in system is small, which was also the outcome of the inferential analysis.

The number of realised donors has nevertheless increased. This partly reflects increasing donation after euthanasia. In 2024, an exceptionally high number of potential donors, despite declining intensive care deaths, also contributed to a high number of realised donors. Greater opportunities for extended criteria donors, abdominal normothermic regional perfusion for DCD donors, machine preservation, and, since 2021, DCD heart donation have further expanded the potential donor pool. Given the limited increase in overall consent, the increase in realised donors cannot be attributed to the opt-out system alone.

A positive aspect of the Dutch system is that all 14 million adults are now registered, ensuring that family approaches are based on the potential donor’s documented wishes. Under the opt-in system, families often faced donation decisions without knowing their loved one’s preferences. The opt-out system has also prompted discussion within many families, with some now initiating donation conversations in intensive care.

#### England

Faced with falling donor numbers in 2024, the Organ Donation Joint Working Group (ODJWG), jointly chaired by the Department of Health and Social Care and NHS Blood and Transplant produced *A Bolder, Braver Approach for Organ Donation in the UK*. [10]. The Group brought together UK clinical leaders, donor family representatives and international experts. It concluded that deemed consent legislation is positive and permissive, does not require amendment and has helped signal support for donation and enable system improvements. However, it found no reliable evidence that opt-out legislation alone increases consent rates and noted persistent public confusion about deemed consent. The report advises against using legislation in public-facing marketing, unless legally required, or in discussions with grieving families. Deemed consent was considered too complex and potentially counterproductive in acute grief, with concern that referencing the law may provoke resistance or “reactance.” Instead, communication should focus on the individual’s values, the positive impact of donation and supporting families. The Group recommends acting within the law but not leading with it, positioning legislation as a supportive framework rather than a tool of persuasion. Implementation of the Report is ongoing across the UK.

England’s experience provides several lessons. We continue to consider the legislation valuable: it signalled societal and governmental support for donation while respecting individual autonomy through the mechanism of opt-out. It also enabled engagement with faith and community groups, broadening conversations beyond the clinical setting.

However, implementation could have been bolder. Campaigns emphasised choice and positioned opt-in and opt-out equally, rather than articulating a clear pro-donation societal norm. The law alone therefore did not generate a pro-donation culture, a factor that moderates policy effectiveness [11]. If opt-out legislation is intended as both a legal and cultural shift, it requires confident, sustained public messaging that supports pro-donation social norms while respecting individual autonomy. Low public acceptance may otherwise hinder successful implementation [12].

#### The Netherlands

The opt-out system was introduced during the COVID-19 pandemic, limiting opportunities to train intensivists on approaching families about the new “No objection” category. In-person training was not possible, and even trained intensivists might wait months or years before encountering their first case. Van Oosterhout et al. found that donor conversations involve a complex interplay between donor registration and clinician-family interactions [13]. Clinicians’ personal considerations, previous experiences with the family and professional context shaped four distinct approaches to discussing “Yes” or “No objection” registrations, each reflecting increasing family influence by the family on the outcome of the donation decision.

The family’s role is also influenced by awareness and understanding of the opt-out system. Registration of 7 million previously unregistered residents occurred during the COVID-19 pandemic, a period of high mortality fear, that may not have been ideal for considering donation preferences. However, 31% registered “No, I don’t want to be an organ donor” suggesting that those strongly opposed could still record their preference.

Similar findings emerged in Nova Scotia, Canada, where deemed consent was also introduced during the pandemic. Sarti et al. found variable family awareness of the new law and their role in decision-making [14]. Some were optimistic about deemed consent, while others felt that continued family consultation limited its potential impact.

### Limitations

Our study has several limitations. First, implementation of opt-out legislation in both England and the Netherlands coincided with the COVID-19 pandemic, making it difficult to separate the effects of legislative change from wider impacts on healthcare systems, public attitudes and organ donation activity. Second, this observational analysis of national registry and audit data cannot establish causality. Third, organ donation activity is influenced by multiple factors beyond legislation. Fourth, differences in registration systems, data collection methods and consent processes limit direct comparisons between countries. Finally, donor registrations and consent rates may not fully capture wider societal attitudes towards donation.

## Conclusion

Five years on, our earlier conclusions remain broadly valid. Legislation alone has not transformed donation rates. Operational infrastructure, professional training, digital systems and stakeholder engagement remain essential. In both England and the Netherlands, opt-out legislation functioned as an enabler, signalling governmental commitment, unlocking investment, and creating opportunities for engagement with faith and community groups.

We underestimated the extent to which legislative change is symbolic rather than transformative in itself. While opt-out legislation signals a pro-donation stance, it does not automatically create a pro-donation culture. The Dutch experience illustrates this clearly: the new category “No objection” remains challenging, with fewer than half of families supporting donation.

We also underestimated the importance of narrative clarity and cultural positioning. In both countries, public campaigns were deliberately neutral, emphasising individual choice and avoiding pressure to donate. While principled, this limited the opportunity to present donation as an expected and valued social contribution. Legislative reform that aspires to cultural change requires sustained, confident public messaging that supports donation as a shared societal good while respecting individual autonomy.

Taken together, the experiences of England and the Netherlands suggest that opt-out legislation should be understood not as a standalone solution, but as a structural platform from which supportive systems, public engagement and a pro-donation culture can be built.

## Data Availability

The original contributions presented in the study are included in the article/Supplementary Material, further inquiries can be directed to the corresponding author.

## References

[1] Parsons JA . Deemed consent for organ donation: a comparison of the English and Scottish approaches. J L Biosciences (2021) 8(1):lsab003. 10.1093/jlb/lsab003 33981445 PMC7989506

[2] NHS Blood and Transplant. Annual activity report 2024/25. ODT clinical – NHS blood and transplant (2025). Available online at: https://www.odt.nhs.uk/statistics-and-reports/annual-activity-report/ (Accessed March 3, 2026).

[3] Jansen NE Williment C Haase-Kromwijk BJJM Gardiner D . Changing to an opt out system for organ donation-reflections from England and Netherlands. Transpl Int (2022) 35(Jul 4):35. 10.3389/ti.2022.10466 35859668 PMC9290126

[4] Office for National Statistics. Office for National Statistics. UK Government (2026). Available online at: https://www.gov.uk/government/organisations/office-for-national-statistics (Accessed March 3, 2026).

[5] Donorregister. Cijfers Newport: Office for National Statistics (2026). Available online at: https://www.donorregister.nl/over-het-donorregister/cijfers (Accessed March 3, 2026).

[6] Nederlandse Transplantatie Stichting. Cijfers over organen en weefsels (Power BI dashboards) (2026). Available online at: https://www.transplantatiestichting.nl/cijfers-over-organen-en-weefsels (Accessed March 3, 2026).

[7] McLaughlin L Williams L Noyes J Al-Haboubi M Boadu P Bostock J Evaluation of the Organ Donation (Deemed Consent) Act, 2019 in England: Final Report. London: National Institute for Health Research, Policy Innovation and Evaluation Research Unit (2024). Report to Department of Health and Social Care and. 10.17037/PUBS.04673056

[8] McLaughlin L Mays N Al-Haboubi M Williams L Bostock J Boadu P Potential donor family behaviours, experiences and decisions following implementation of the organ donation (deemed consent) act 2019 in England: a qualitative study. Intensive Crit Care Nurs (2025) 86:103816. 10.1016/j.iccn.2024.103816 39217721

[9] YouGov. How Good or Bad Are National NHS Services? (2026). Available online at: https://yougov.com/en-gb/trackers/how-good-or-bad-are-national-nhs-services (Accessed March 3, 2026).

[10] NHS Blood and Transplant. A bolder, braver approach for organ donation in the UK: final report of the organ donation joint working group (2025). Available online at: https://nhsbtdbe.blob.core.windows.net/umbraco-assets-corp/38066/odjwg-report.pdf (Accessed March 3, 2026).

[11] Johnson EJ Goldstein D . Medicine. Do defaults save lives? Science (2003) 302(5649):1338–9. 10.1126/science.1091721 14631022

[12] Güntürkün P Studte S Winkler D Clement M Tan JHW Merz EM Crowding-out effects of opt-out defaults: evidence from organ donation policies. PNAS Nexus (2025) 4(10):pgaf311. 10.1093/pnasnexus/pgaf311 41163788 PMC12560092

[13] van Oosterhout SPC van der Niet AG Abdo WF Boenink M Cherpanath TGV Epker JL How clinicians discuss patients' donor registrations of consent and presumed consent in donor conversations in an opt-out system: a qualitative embedded multiple-^cas44e^ study. Crit Care (2023) 27(1):299. 10.1186/s13054-023-04581-9 37507800 PMC10375668

[14] Sarti AJ Sutherland S Weiss M Landry A Hemming H Dirk J Nova Scotia's deemed consent for deceased organ donation: family member perspectives and experiences in the ICU setting. Transpl Direct (2024) 10(11):e1713. 10.1097/TXD.0000000000001713 39399063 PMC11469852

